# PSQAN: a pipeline to prioritize novel and biologically relevant transcripts from long-read RNA sequencing

**DOI:** 10.1093/bioadv/vbaf293

**Published:** 2025-11-20

**Authors:** Siddharth Sethi, Emil K Gustavsson, Harpreet Saini, Mina Ryten

**Affiliations:** Astex Pharmaceuticals, Cambridge, CB4 0QA, United Kingdom; Department of Genetics and Genomic Medicine, Great Ormond Street Institute of Child Health, University College London, London, WC1N 1EH, United Kingdom; Department of Genetics and Genomic Medicine, Great Ormond Street Institute of Child Health, University College London, London, WC1N 1EH, United Kingdom; Dementia Research Institute, Department of Clinical Neuroscience, University of Cambridge, Cambridge, CB2 0AH, United Kingdom; Astex Pharmaceuticals, Cambridge, CB4 0QA, United Kingdom; Department of Genetics and Genomic Medicine, Great Ormond Street Institute of Child Health, University College London, London, WC1N 1EH, United Kingdom; Dementia Research Institute, Department of Clinical Neuroscience, University of Cambridge, Cambridge, CB2 0AH, United Kingdom; Department of Clinical Neurosciences, School of Clinical Medicine, University of Cambridge, Cambridge, CB2 0SP, United Kingdom; Academic Department of Medical Genetics, School of Clinical Medicine, University of Cambridge, Cambridge, CB2 0SP, United Kingdom

## Abstract

**Motivation:**

Long-read RNA sequencing has the potential to accurately quantify transcriptomes and reveal the isoform diversity of disease-causing genes. However, despite the recent advances in analysis tools for transcript discovery, long-read RNA sequencing data is still challenging to analyse, due to the detection of hundreds or even thousands of novel transcripts per gene.

**Results:**

Here, we introduce PSQAN, a workflow to help researchers prioritize high-confidence and potentially biologically relevant transcripts associated with candidate genes and make transcript characterization results more interpretable. PSQAN performs a gene-based analysis on characterized transcripts generated by SQANTI3 and TALON. PSQAN re-groups transcripts into easily interpretable categories to facilitate their prioritization, allows transcript-level expression thresholds, and generates visualizations to determine optimal expression thresholds. Overall, we demonstrate that PSQAN is a useful tool which enables users to identify known and novel transcripts of potential biological importance.

**Availability and implementation:**

PSQAN is an analysis workflow implemented in Snakemake and R and is licensed under the GNU General Public License version 3. The source code and documentation of this tool is available at https://github.com/sid-sethi/PSQAN.

## 1 Introduction

Long-read RNA sequencing (lrRNA-seq) has emerged as a powerful tool to accurately measure isoform proportions and identify novel isoforms, tasks which are highly challenging with short-read RNA sequencing. The rising popularity of lrRNA-seq has led to the development of several tools for transcript discovery, quantification, and characterization, such as TALON (Wyman *et al.* 2020), StringTie2 (Kovaka *et al.* 2019), IsoQuant (Prjibelski *et al.* 2023), SQANTI3 (Pardo-Palacios *et al.* 2024a), and Swan (Reese and Mortazavi 2021). Despite the advances in tools to process lrRNA-seq data, the downstream analysis of transcriptional data remains challenging due to the detection of thousands of novel transcripts (Tian *et al.* 2021, Zhang *et al.* 2022, Gustavsson *et al.* 2024) and the lack of tools to prioritize functionally important transcripts. For instance, Gustavsson *et al.* (2024) performed targeted Pacific Biosciences isoform sequencing (Iso-Seq) for *GBA1* and *GBAP1* across 12 brain regions, and identified 2368 and 3083 unique transcripts for *GBA1* and *GBAP1*, respectively, each supported by at least two full-length reads. From such a large number of transcripts, it is difficult to distinguish between stable transcripts of potential biological importance, partially processed RNAs and splicing noise (García-Ruiz *et al.* 2025). Furthermore, when using lrRNA-seq to identify rare and novel transcripts, the recommendation is to incorporate multiple replicates in the study design and implement transcript-level filters ([Bibr vbaf293-B7][Bibr vbaf293-B8]). However, determining optimal expression thresholds for filtering and selecting transcripts which are reproducible across samples remains a significant challenge. Consequently, researchers find it challenging to interpret lrRNA-seq data effectively and generate relevant hypothesis which could be experimentally validated in the laboratory.

Here, we present **P**ost-transcriptomic **S**tructural **Q**uality **A**ssessment and **N**ormalization (PSQAN); a workflow designed to help researchers identify high-confidence transcripts associated with candidate genes. PSQAN performs a gene-based analysis on characterized transcripts generated by SQANTI3 and TALON. PSQAN normalizes transcript expression per gene and re-groups transcripts into actionable categories to support transcript prioritization, hence making the results more interpretable. PSQAN generates visualizations to help users determine optimal expression thresholds for detecting both known and novel transcripts of probable biological importance. Furthermore, PSQAN allows users to apply multiple transcript level expression thresholds, both to per sample and across all samples. Lastly, PSQAN generates visualizations and an HTML report, enabling users to explore the known and novel transcripts expressed by a gene, alongside their transcript categories and transcript expression.

## 2 Description

### 2.1 Input data

**Figure 1.**
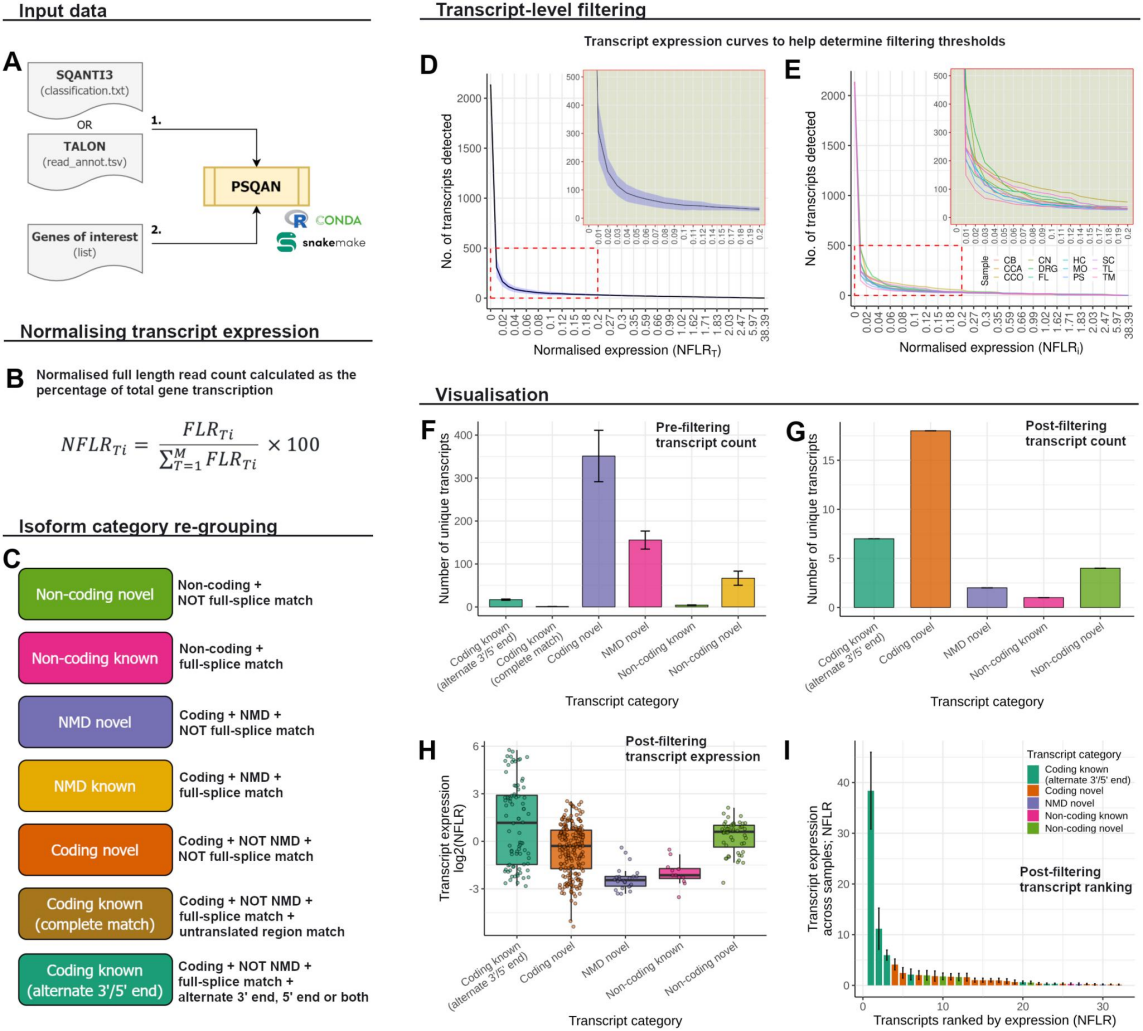
Overview of the PSQAN workflow. (A) A schematic showing the input data required by PSQAN. (B) Normalization of transcript expression performed by PSQAN for each gene. (C) Description of the isoform categories generated by PSQAN. Panels (D–I) show example plots produced by PSQAN for each gene. These examples were generated from targeted Pacific Biosciences Iso-Seq data for *GBA1* across 12 brain regions. (D) Plot showing the number of transcripts detected as a function of varying expression thresholds. The blue shaded area shows the standard deviation across the samples. The inset plot at the top right shows a magnified view of the region highlighted in red. (E) Plot showing the number of transcripts detected as a function of varying expression thresholds for each individual sample. The inset plot at the top right shows a magnified view of the region highlighted in red. CN: caudate nucleus; CB: cerebellum; CCO: cerebral cortex; CCA: corpus callosum; DRG: dorsal root ganglion; FL: frontal lobe; HC: hippocampus; MO: medulla oblongata; PS: pons; SC: spinal cord; TL: temporal lobe; TM: thalamus. (F) Bar plot showing the number of transcripts detected before applying filtering based on expression thresholds. The bar represents the mean across samples, and the error bars show the standard deviation. (G) Bar plot showing the number of transcripts detected after applying PSQAN’s filtering based on expression thresholds. (H) Boxplot showing the expression of transcripts in each transcript category. Each data point represents a transcript per sample. (I) Bar plot showing all identified transcripts ranked according to their normalized expression. The bar represents mean expression across samples, and the error bars show the standard deviation.

PSQAN is a workflow designed to perform a gene-based analysis on lrRNA-seq data, post transcript characterization (Fig. 1). PSQAN can be used with the transcript characterization output of either SQANTI3 or TALON, which are the two most prominently used tools in lrRNA-seq analysis. PSQAN takes the output produced by SQANTI3 or TALON as input, along with a list of candidate genes to analyse (Fig. 1A, see Section 1.1, available as supplementary data at *Bioinformatics Advances* online for more details).

### 2.2 Normalizing transcript expression per gene

Transcript expression in lrRNA-seq data is usually quantified as the number of full-length reads (FLRs) associated with a transcript. However, it is difficult for researchers to associate this FLR count with the total gene expression. For instance, consider a gene with five transcripts—A, B, C, D and E; having FLR counts of 40, 20, 10, 10, and 10, respectively. While it is evident that transcript A is the most abundant based on FLR count, this metric alone does not convey that A represents only ∼45% (40/90) of the total gene expression. This implies that the remaining transcripts, although individually less abundant, collectively account for ∼55% of the expression, highlighting their potential biological significance. To address this, PSQAN calculates the normalized full-length reads for each transcript (${\mathrm{NFLR}}_{Ti}$), which quantifies transcript expression as the percentage of total gene transcription (Fig. 1B). This normalization emphasizes transcript usage relative to overall gene output, thereby simplifying interpretation. For instance, a transcript with an NFLR value of 10.0 would imply that it accounts for 10% towards all transcripts generated from the gene locus. PSQAN’s normalization also removes variation due to overall gene expression differences between samples, hence making comparisons of transcript usage independent of absolute gene expression (see Section 1.2, available as supplementary data at *Bioinformatics Advances* online for more details).

### 2.3 Isoform category re-grouping

If PSQAN is used with the output of SQANTI3, it also performs isoform re-grouping into categories which are easy to interpret and facilitates prioritizing potentially relevant transcripts. Using the open reading frame prediction, nonsense-mediated decay (NMD) prediction and structural categorization (based on the comparison with reference annotation) of SQANTI3, PSQAN groups the identified isoforms into the following seven categories: non-coding novel, non-coding known, NMD novel, NMD known, coding novel, coding known (complete match) and coding known (alternate 3′/5′ end) (Fig. 1C, see Section 1.3, available as supplementary data at *Bioinformatics Advances* online for more details).

### 2.4 Transcript-level filtering

A common strategy to mitigate false-positives in lrRNA-seq data and to identify high-confidence transcripts is to generate data across several samples. For novel transcript discovery, only transcript models that are reproducible across samples and expressed above a minimum threshold in each sample should be retained. Existing tools like SQANTI3 and TALON, can be used to filter transcripts which are not expressed above a given threshold in all samples. However, such filtering alone is often insufficient (see Section 1.4, available as supplementary data at *Bioinformatics Advances* online for more details), and PSQAN extends this approach by implementing two additional filtering strategies which provides more flexibility and data-driven refinement: (i) a minimum threshold on the mean expression across all samples (${\mathrm{NFLR}}_{T}$); and (ii) a minimum percentage of samples in which a transcript must meet the minimum per sample expression threshold. Furthermore, to aid researchers, PSQAN provides a visualization of the number of detected transcripts as a function of varying expression thresholds, showing the number of transcripts which will be retained at every ${\mathrm{NFLR}}_{T}$ threshold (Fig. 1D). At lower expression thresholds, the number of detected transcripts is very high. This plot enables users to visually inspect and determine an appropriate ${\mathrm{NFLR}}_{T}$ threshold for retaining high-confidence transcripts. In datasets with multiple samples, PSQAN generates a ${\mathrm{NFLR}}_{Ti}$ curve per sample (Fig. 1E), allowing researchers to examine the variability in transcript detection across samples. This visualization supports informed decision-making regarding: (i) the minimum expression value at which a transcript should be expressed within each sample; and (ii) the minimum number of samples in which a transcript must meet the minimum expression threshold to be considered reproducible.

### 2.5 Visualization

PSQAN generates multiple visualizations to aid in the interpretation of results. In addition to visualizing transcripts across varying expression thresholds (Fig. 1D and E), PSQAN plots the number of transcripts detected in each isoform category and their normalized expression, both before and after filtering (Fig. 1F–H). For datasets with multiple samples, PSQAN also computes variability across the samples as standard deviation, which is displayed as error bars. PSQAN displays all transcripts associated with a gene ranked by their normalized expression and coloured by transcript category (Fig. 1I), allowing users to easily identify dominant transcripts of a gene. Lastly, PSQAN provides an option to generate a gene-level HTML report, compiling all visualizations to facilitate result interpretation.

## 3 Results

To evaluate PSQAN, we retrieved transcripts characterized by SQANTI3 for lrRNA-seq data of the human WTC11 cell line, generated using PacBio cDNA sequencing platform as a part of the Long-read RNA-seq Genome Annotation Assessment Project (LRGASP) ([Bibr vbaf293-B7][Bibr vbaf293-B8]). From the SQANTI3 output, we extracted transcripts associated with 1000 randomly selected genes (Table 1, available as supplementary data at *Bioinformatics Advances* online) and applied the PSQAN pipeline with default parameters to prioritize transcripts.

**Figure 2.**
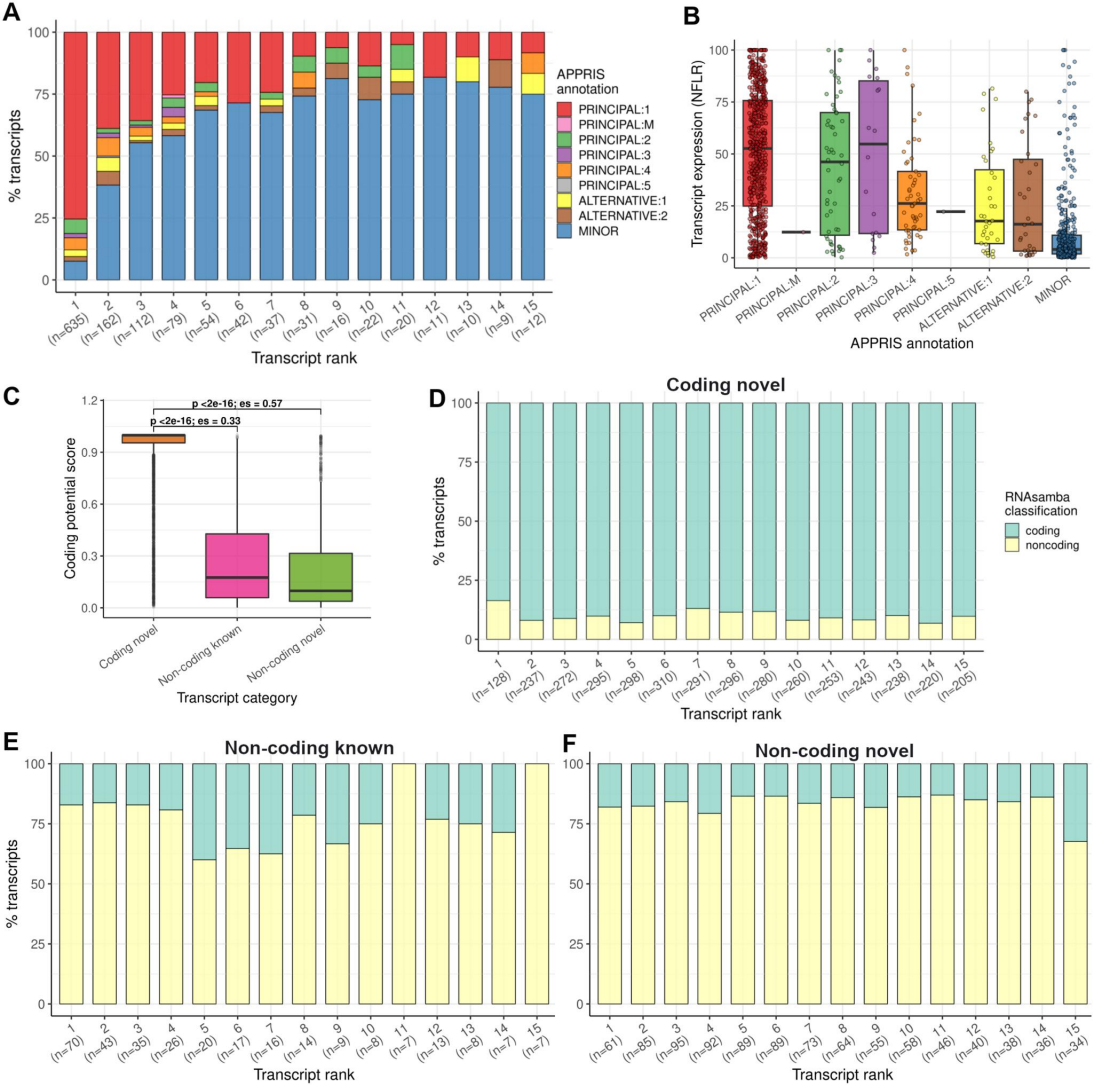
Evaluation of PSQAN. (A) Bar chart showing the overlap between transcripts in the ‘coding known’ group (defined as those with complete match or alternate 3′/5′ ends) and APPRIS annotation, across the top 15 transcripts ranked by PSQAN. Each bar represents the percentage of ‘coding known’ transcripts at a given rank that matches the APPRIS annotation. (B) Distribution of transcript expression levels across APPRIS annotation categories. (C) Boxplots comparing the coding potential scores of ‘coding novel’ transcripts with the negative control set (‘non-coding known’ and ‘non-coding novel’), as predicted by RNAsamba. *P: P* value of comparison calculated using two-sided Wilcoxon rank-sum test; es: Wilcoxon effect size (r). (D–F) Bar charts showing the RNAsamba classification results for ‘coding novel’, ‘non-coding known’ and ‘non-coding novel’ groups across the top 15 transcripts ranked by PSQAN.

To assess the biological relevance of known transcripts prioritized by PSQAN, we compared them with annotations from the APPRIS (Rodriguez *et al.* 2022) database, which designates principal isoforms (‘PRINCIPAL:1’ or ‘PRINCIPAL: M’) for each protein-coding gene, based on protein structure, function features, and cross-species conservation. For each gene, we evaluated the transcripts in the ‘coding known’ group (i.e. those with complete match or alternate 3’/5’ ends) among the top 15 transcripts ranked by PSQAN. Among the transcripts ranked first, 75% (479/635) matched the APPRIS principal isoform (‘PRINCIPAL:1’), followed by 39% (63/162) at rank 2 and 36% (40/112) at rank 3 (Fig. 2A, Table 2, available as supplementary data at *Bioinformatics Advances* online). This percentage declined to 8% (1/12) at rank 15. These results indicate that PSQAN effectively prioritizes known transcripts, as evidenced by the high concordance (75%) between top-ranked ‘coding known’ transcripts and APPRIS principal isoforms, which represent the main cellular isoforms of protein-coding genes (Rodriguez *et al.* 2022). The decline in concordance with lower ranks further suggests that transcripts ranked lower are less likely to be the principal isoform. We also took this opportunity to compare the expression of transcripts within every APPRIS annotation category. We observed that principal isoforms contribute, on average, only ∼51% (NFLR = 50.82) towards the total transcription of their associated genes (Fig. 2B), highlighting the potential functional relevance of other, less abundant transcripts. This observation is consistent with previous studies that reported the absence of a single dominant transcript associated with the gene (Evans *et al.* 2024, Gustavsson *et al.* 2024, Minnis *et al.* 2025).

Next, we assessed the relevance of novel transcripts prioritized by PSQAN by evaluating their coding potential. For each gene, we analysed transcripts in the ‘coding novel’ group among the top 15 transcripts ranked by PSQAN, using RNAsamba (Camargo *et al.* 2020) - a neural network-based classification model which predicts the coding potential of RNA sequences. To provide a baseline for comparison, transcripts classified as ‘non-coding known’ and ‘non-coding novel’ were used as a negative control set in this analysis. We found that ‘coding novel’ transcripts exhibited significantly higher coding potential scores compared to ‘non-coding known’ ($P<2.2\times 10^{-16}$, effect size (es) = 0.33, two-sided Wilcoxon Rank Sum Test) and ‘non-coding novel’ ($P<2.2\times 10^{-16}$, es = 0.57, two-sided Wilcoxon Rank Sum Test) transcripts (Fig. 2C). On average, across the top 15 ranks, 90% of ‘coding novel’ transcripts were classified as potentially ‘coding’ (Fig. 2D), in contrast to 26% and 17% for the ‘non-coding known’ (Fig. 2E) and ‘non-coding novel’ (Fig. 2F) transcripts, respectively (Table 3, available as supplementary data at *Bioinformatics Advances* online). These results suggest that PSQAN effectively prioritizes novel transcripts with strong coding potential (see Section 2, available as supplementary data at *Bioinformatics Advances* online for more case-studies).

## 4 Conclusions

In conclusion, PSQAN provides a platform to perform a downstream gene-level analysis of lrRNA-seq data, facilitating prioritization of transcripts with potential biological importance. PSQAN’s normalization methodology, isoform categorization and visualizations make the data more interpretable. It also enables users to implement transcript-level expression thresholds, which can help identify high-confidence novel transcripts.

## Supplementary Material

vbaf293_Supplementary_Data

## Data Availability

Transcripts characterized by SQANTI3 for lrRNA-seq data of the human WTC11 cell line were downloaded from https://conesalab.uv.es/SQANTI3. APPRIS data was downloaded from the portal https://appris.bioinfo.cnio.es (species: ‘human’; Gene Dataset: ‘Gencode48/Ensembl114’). The data presented in this study is provided in the Supplementary Data.
