# Supplementary material for: PSQAN: a pipeline to prioritize novel and biologically relevant transcripts from long-read RNA sequencing
Source: Bioinform Adv. 2025 Nov 20;5(1):vbaf293. doi: 10.1093/bioadv/vbaf293 (PMC12701792; doi:10.1093/bioadv/vbaf293)
Supplement: vbaf293_Supplementary_Data [file vbaf293_supplementary_data.zip › Supplementary Materials.docx]

**Supplementary Materials**

**PSQAN: a pipeline to prioritise novel and biologically relevant transcripts from long-read RNA sequencing**

#### Siddharth Sethi^1,2*^, Emil K. Gustavsson^2,3^, Harpreet Saini^1^, Mina Ryten^2,3,4,5*^

1. Astex Pharmaceuticals, 436 Cambridge Science Park, Cambridge, UK.
2. Department of Genetics and Genomic Medicine, Great Ormond Street Institute of Child Health, University College London, London, UK.
3. Dementia Research Institute, Department of Clinical Neuroscience, University of Cambridge, Cambridge UK.
4. Department of Clinical Neurosciences, School of Clinical Medicine, University of Cambridge, Cambridge, UK.
5. Academic Department of Medical Genetics, School of Clinical Medicine, University of Cambridge, Cambridge, UK

* Corresponding author: Dr Siddharth Sethi ([Siddharth.sethi@astx.com](mailto:Siddharth.sethi@astx.com)) and Professor Mina Ryten ([mr2022@medschl.cam.ac.uk](mailto:mr2022@medschl.cam.ac.uk))

1. **Supplementary Methods**
   1. **Input data**

PSQAN can be used with the transcript characterisation output of either SQANTI3 or TALON. SQANTI3 performs isoform classification by comparing splice junctions to a reference annotation. It can also incorporate orthogonal data - such as, CAGE peaks, polyA motifs and splice junctions from short-read data, to calculate various transcript-level descriptors for quality control. SQANTI3 also allows users to filter out potential false positive isoforms, such as those resulting from internal priming events (indicated by a high percentage of genomic "A"s in the 20 bp downstream window) or with junctions that may be artifacts of template switching. TALON, like SQANTI3, categorises isoforms into known and novel sub-categories by comparing them to a reference annotation, and flags false positives isoforms resulting from internal priming events.

PSQAN takes the output produced by SQANTI3 or TALON as input, along with a list of candidate genes to analyse. For each gene, PSQAN extracts the isoforms associated with the gene from the output generated by SQANTI3/TALON. Since the filtering steps in SQANTI3 and TALON are optional and may be skipped, PSQAN applies its own filtering criteria prior to processing to ensure the removal of potential genomic contamination and rare PCR artifacts. PSQAN removes isoforms with a high percentage of genomic "A"s in their downstream 20 bp window (80% is the default), or if one of its junctions is predicted to be a template switching artifact (tagged as "RTS_stage" by SQANTI3).

It should be noted that the output of TALON does not contain all the transcript-level descriptors required by PSQAN. As a result, certain PSQAN processes are skipped when using TALON output. The processes performed by PSQAN for SQANTI3 and TALON are summarised in Table 1.

**Table 1: Summary of processes performed by PSQAN for the output of SQANTI3 and TALON**

| **PSQAN process** | **SQANTI3** | **TALON** |
| --- | --- | --- |
| Filtering internal priming artifacts | Yes | Yes |
| Filtering template switching artifacts | Yes | No (missing required data) |
| Normalising transcript expression | Yes | Yes |
| Isoform re-categorisation | Yes | No (missing required data) |
| Transcript-level filtering | Yes | Yes |
| Visualisations | Yes | Yes |

- 1. **Normalising transcript expression per gene**

PSQAN calculates the normalised full-length reads (NFLR) for each transcript, which quantifies transcript expression as the percentage of total gene transcription. This normalisation emphasizes transcript usage relative to overall gene output, thereby simplifying interpretation. PSQAN’s normalisation also removes variation due to overall gene expression differences between samples, hence making comparisons of transcript usage independent of absolute gene expression. However, this approach has certain limitations that should be considered. First, because this metric does not account for sequencing depth, it may introduce bias if samples with very low coverage are analysed alongside high-coverage samples. Second, it may not be suitable on its own for differential expression analysis at either the gene or transcript level. Third, transcript usage derived from this metric cannot be compared across different genes, as each gene is normalised independently.

Given a transcript $\boldsymbol{T}$ in sample $\boldsymbol{i}$ with $\boldsymbol{FLR}$ as the number of full-length reads mapped to the transcript $\boldsymbol{T}$, PSQAN calculates the normalised full-length reads ($\boldsymbol{NFLR}_{\boldsymbol{Ti}}$) as:

$$\boldsymbol{NFLR}_{\boldsymbol{Ti}}= \frac{\boldsymbol{FLR}_{\boldsymbol{Ti}}}{\sum_{\boldsymbol{T}=\mathbf{1}}^{\boldsymbol{M}} \boldsymbol{FLR}_{\boldsymbol{Ti}}} \times\mathbf{100}$$

where, $\boldsymbol{NFLR}_{\boldsymbol{Ti}}$ represents the normalised full-length read count of transcript $\boldsymbol{T}$ in sample $\boldsymbol{i}$, $\boldsymbol{FLR}_{\boldsymbol{Ti}}$ is the full-length read count of transcript $\boldsymbol{T}$ in sample $\boldsymbol{i}$, and $\boldsymbol{M}$ is the total number of transcripts identified to be associated with the gene after filtering. Finally, to summarise the expression of a transcript associated with a gene across multiple samples (if any), PSQAN calculates the mean of $\boldsymbol{NFLR}_{\boldsymbol{Ti}}$ across all the samples:

$$\boldsymbol{NFLR}_{\boldsymbol{T}}= \frac{\sum_{\boldsymbol{i}=\mathbf{1}}^{\boldsymbol{N}} \boldsymbol{NFLR}_{\boldsymbol{Ti}}}{\boldsymbol{N}}$$

where, $\boldsymbol{NFLR}_{\boldsymbol{T}}$ represents the mean expression of transcript $\boldsymbol{T}$ across all samples and $\boldsymbol{N}$ is the total number of samples.

- 1. **Isoform category re-grouping**

Using the open reading frame (ORF) prediction, nonsense-mediated decay (NMD) prediction and structural categorisation (based on the comparison with reference annotation) of SQANTI3, PSQAN groups the identified isoforms into the following seven categories: (1) non-coding novel - if predicted to be non-coding and not a full-splice match with the reference; (2) non-coding known - if predicted to be non-coding and a full-splice match with the reference; (3) NMD novel - if predicted to be coding and NMD, and not a full-splice match with the reference; (4) NMD known - if predicted to be coding and NMD, and a full-splice match with the reference; (5) coding novel - if predicted to be coding and not NMD, and not a full-splice match with the reference; (6) coding known (complete match) - if predicted to be coding and not NMD, and a full-splice and untranslated region match with the reference; and (7) coding known (alternate 3'/5' end) - if predicted to be coding and not NMD, and a full-splice match with the reference but with an alternate 3’ end, 5’ end or both 3’ and 5’ end.

- 1. **Transcript-level filtering**

Existing tools like SQANTI3 and TALON, can be used to filter transcripts which are not expressed above a given threshold in all samples. However, such filtering alone is often insufficient, and PSQAN addresses several limitations not handled by existing tools: (1) **Reproducibility across samples:** when multiple samples are available, applying the same threshold uniformly across all samples can be overly strict. In practice, it may be more informative to retain transcripts that meet the threshold in a subset of samples, reflecting reproducibility rather than absolute presence; (2) **Mean expression filtering:** another practical scenario is filtering based on the average expression across all samples, rather than a per-sample threshold. This approach can help retain transcripts with moderate but consistent expression levels; and (3) **Lack of guidance for threshold selection:** determining an appropriate minimum expression threshold to remove artefacts is non-trivial as the threshold can vary across datasets, and neither SQANTI3 nor TALON provide tools or guidance to assist users in choosing dataset-specific thresholds.

1. **Supplementary Results**

PSQAN was used to analyse and interpret the lrRNA-seq dataset generated in our previous work [1-4]. To demonstrate PSQAN’s utility, we describe the results from Gustavsson *et al.,*[1], which included targeted Pacific Biosciences Iso-Seq data for *GBA1* across 12 brain regions. The isoforms detected from this data were characterised and classified using SQANTI3, which resulted in the identification of 2,368 *GBA1* unique transcripts (Fig. 1D-F). Using PSQAN’s ${NFLR}_{T}$ threshold curves (Fig. 1D and E), we decided on the following criteria to remove low-confidence transcripts: (1) transcript expression per sample should be at least 0.1% of total transcription, i.e., ${NFLR}_{Ti}\geq0.1$; (2) transcript should pass the ${NFLR}_{Ti}$ threshold in at least 80% of total samples; and (3) transcript expression across samples should be at least 0.3% of total transcription, i.e., ${NFLR}_{T}\geq0.3$. Overall, application of PSQAN on this data identified a total of 32 *GBA1* transcripts (Fig. 1G), of which, 18 *GBA1* transcripts were categorised as ‘coding novel’. Using PSQAN’s transcript ranking (Fig. 1I), we observed that the most highly expressed transcript of *GBA1*, categorised as coding known (alternate 3’/5’ end), only contributed ~38% towards the total gene transcription.

To validate the ‘coding novel’ ORFs of *GBA1*, we investigated their presence in an independent dataset comprised of targeted Iso-Seq of *GBA1* in iPSC-derived cortical neurons (n = 6), astrocytes (n = 3), and microglia (n = 3). We were able to detect, and hence, validate all the ‘coding novel’ ORFs of *GBA1* in this independent dataset [1]. We further tested the most highly expressed novel coding ORFs of *GBA1* (n = 3) by cloning them into a vector with a C-terminal FLAG-tag. We transfected these vectors into H4 cells with homozygous knockout of *GBA1* and found translation of all transcripts (see [1] for more details).

1. **References**

1. Gustavsson, E.K., et al., The annotation of GBA1 has been concealed by its protein- coding pseudogene GBAP1. Sci Adv, 2024. 10(26): p. eadk1296.

2. Evans, J.R., et al., The diversity of SNCA transcripts in neurons, and its impact on antisense oligonucleotide therapeutics. bioRxiv, 2024: p. 2024.05.30.596437.

3. Gustavsson, E.K., et al., Independent Generation of Amyloid-β via Novel APP Transcripts. bioRxiv, 2025: p. 2025.05.13.651054.

4. Minnis, C.J., et al., Targeted long-read RNA sequencing reveals the complexity of CLN3 transcription and the consequences of the most common 1-kb deletion in patients with juvenile CLN3 disease. bioRxiv, 2025: p. 2025.04.24.650398.
